# Psychometric properties of the French version of the climate change worry scale

**DOI:** 10.1016/j.joclim.2024.100361

**Published:** 2024-11-13

**Authors:** Sarah Shepherd, Patrick Raynal, Myriam Guedj

**Affiliations:** Centre d'Etudes et de Recherches en Psychopathologie et Psychologie de la Santé, Université de Toulouse, France

**Keywords:** Climate change worry, Climate change anxiety, Eco-anxiety

## Abstract

**Introduction:**

As concern over climate change keeps growing, there is a need for reliable tools to assess the psychological impact of this global issue across different languages. This study presents the first French adaptation of the Climate Change Worry Scale (CCWS) and evaluates its psychometric properties. The CCWS, originally developed in English, is a 10-item self-report measure assessing personal worry about climate change.

**Methods:**

A total of 442 participants (82.1% female, mean age = 32.45, SD = 12.50) completed the CCWS along with the Climate Change Anxiety Scale (CCAS), the Penn State Worry Questionnaire (PSWQ), and the Depression Anxiety Stress Scales (DASS).

**Results:**

The CCWS showed robust internal consistency, with Cronbach's alpha and McDonald's omega values of 0.91. Convergent validity was supported by a strong correlation between the CCWS and CCAS (*r* = 0.79). Divergent validity was shown by weaker correlations with general worry assessed with PSWQ (*r* = 0.31) and symptoms of depression, anxiety, and stress measured with DASS (*r* = 0.24–0.30). An exploratory factor analysis supported a one-factor solution for the CCWS, explaining 51% of the variance. Factor loadings of the ten items ranged from 0.61 to 0.82. A subsequent confirmatory factor analysis confirmed an adequate fit for a reduced six-item version of the scale.

**Conclusions:**

These findings suggest that the French version of the CCWS is a reliable and valid tool for measuring climate change worry. Its strong psychometric properties make it suitable for use in French-speaking populations, enabling future cross-cultural research on climate-related psychological impacts.

## 1 Introduction

Climate change is an urgent global challenge with significant implications for public health [1,2], including heat-related illnesses and mortality, food and water security, spread of infectious diseases, air quality and respiratory issues, and mental health impacts [[3], [4], [5]]. Climate change poses risks to mental health, with studies showing that short-term exposure to extreme weather, warming, and cyclones can worsen mental health [6]. Mental health can be also altered by indirect consequences of climate changes, such as loss of land, forced migration, and changes in social, ecological, economic or cultural environments [7,8]. Additionally, growing awareness of the existential threats posed by climate change is increasingly affecting individuals' wellbeing and mental health, particularly among children and young people [2,8]. A survey of 10,000 individuals aged 16–25 across five continents found that a majority were very or extremely concerned about climate change [9].

People's perceptions about climate change are associated with various psychological responses, including climate change anxiety (also called ecological anxiety or eco-anxiety) and climate change worry (CCW) [10,11]. Eco-anxiety can be defined as a distressing psychological condition characterized by emotional (such as fear and anger), cognitive (like trouble concentrating), physiological (e.g., muscle tension), and behavioral (such as sleep disturbances) symptoms [12,13]. It arises from the awareness of current ecological crises, including anthropogenic climate change, leading to a pervasive sense of helplessness, grief, and existential dread about the impact of human actions on the environment and the well-being of future generations [10,12,13]. In contrast, CCW refers to persistent thoughts about climate change and its consequences without the explicit presence of emotional, physiological, or behavioral symptoms typically associated with climate anxiety [11,14]. CCW can be viewed as a verbal-linguistic process involving the generation of ruminative thoughts and concern about the climate [11,14], a process that appears to be more cognitive in nature compared to climate anxiety. Authors suggested that CCW serves as an initial stage in the coping process, while climate anxiety can be seen as a potential outcome of prolonged CCW [14,15]. A recent study across six European countries found that CCW was negatively related with mental well-being [16].

A promising tool for assessing CCW is the Climate Change Worry Scale (CCWS), designed to assess proximal worry about climate change rather than social or global impacts [11]. The CCWS was initially developed in English and is composed of ten items (e.g., "I worry about how climate change may affect the people I care about"; "Thoughts about climate change cause me to have worries about what the future may hold"). Responses are given on a 5-point scale ranging from 1 ("never") to 5 ("always"), reflecting how often individuals experience worries related to climate change. All items are intended to be combined into a total score, with higher scores indicating a greater level of concern [11].

Following the development of the initial English version, Italian [17], Polish [14] and Slovenian [18] adaptations of the CCWS have been introduced. All four versions showed strong psychometric properties, including high internal consistency (Cronbach's alpha and McDonald's omega values ≥ .90), despite the Italian version's consistency being calculated without two of its items [11,14,17,18]. Regarding convergent validity, the CCWS showed moderate to strong positive correlations with measures of climate change anxiety or engagement in pro-environmental behaviors, with statistically significant correlations ranging from .52 to .78 [17,18]. In terms of divergent validity, CCWS scores showed a small positive correlation with general worry, with a statistically significant correlation of .17 [11], as well as moderate positive correlations with general anxiety and depression symptoms, with statistically significant correlations around .30, except for the Italian version, which did not reveal any significant association with these symptoms [11,14,17,18]. In terms of internal structure, a one-factor solution emerged as the most suitable model across all four versions [11,14,17,18]. However, in the Italian adaptation, several model fit indices fell below acceptable levels, resulting in the removal of two items to improve fit [17].

The present study aimed to introduce the first French adaptation of the CCWS and to evaluate its psychometric properties. By adapting the scale for French speakers, this research will contribute to a broader understanding of CCW and its psychological impacts, enabling future studies to explore the nuances of CCW across diverse cultural and linguistic settings.

## 2 Materials and methods

### 2.1 Participants

Participants were recruited via an advertisement posted on social networks. The advertisement contained a link to an online questionnaire on LimeSurvey. To access the questionnaire, participants first had to read an information note and provide their consent. To be eligible, participants had to be aged over 18 and speak French fluently. The questionnaires were anonymous and participants did not receive compensation. The data were collected from September 2022 to May 2023. Participants provided information on age, gender, marital status, education, and occupational category. The study protocol was in accordance with the Helsinki Declaration and approved by the local ethics committee (Comité d'Ethique de la Recherche de l'Université de Toulouse) and the Data Protection Officer of the University of Toulouse-Jean Jaurès.

### 2.2 Measures

#### 2.2.1 Climate Change Worry Scale (CCWS)

The CCWS is a self-report questionnaire assessing personal worry about climate change rather than social or global impacts [11]. It is composed of 10 items (e.g., "I worry about climate change more than other people") rated as follows: 1 = never; 2 = rarely; 3 = sometimes; 4 = often; 5 = always. In this current study, the CCWS was translated from English to French using a back translation procedure [19], i.e., the original version was translated into French and then back into English by two different translators. Differences were then discussed in a research meeting to decide the best translation for each item.

#### 2.2.2 Climate Change Anxiety Scale (CCAS)

Anxiety about climate change was measured with a French version of the CCAS [12,20]. This self-report instrument contains 22 items (e.g., "Thinking about climate change makes it difficult for me to concentrate") rated between 1 = "never" and 5 = "almost always". Cronbach's alpha of the French CCAS was 0.86 in a former study [20].

#### 2.2.3 Penn State Worry Questionnaire (PSWQ)

General worry was measured using a French version of the PSWQ, a self-report instrument widely used for assessing generalized anxiety disorder and other disorders characterized by worry [21,22]. It contains 16 items (e.g., "Many situations make me worry") rated from 1 ("not at all typical of me") to 5 ("very typical of me"). Cronbach's alpha of the French PSWQ was 0.92 in a former study [21].

#### 2.2.4 Depression Anxiety Stress Scales (DASS)

Depression, anxiety and stress symptoms were measured using a French translation of the DASS [23,24], a widely-used self-report questionnaire with 42 items assessing the three following dimensions: depression (e.g., "I felt down-hearted and blue"), anxiety (e.g., "I felt I was close to panic") and stress (e.g., "I felt that I was rather touchy"). Items were rated from 0 = "does not apply to me at all" to 3 = "applies to me very much, or most of the time". In a previous study using the French DASS, Cronbach's alpha values were 0.94, 0.88, and 0.91 for depression, anxiety and stress subscales, respectively [23].

### 2.3 Data analysis

Data were screened for incomplete responses, and only complete cases were retained. Univariate outliers were identified using z-scores, leading to the exclusion of three cases where CCAS total scores exceeded the mean by more than 3.29 standard deviations. Multivariate outliers were identified using Mahalanobis distance with a significance level of *p* < .001, leading to the exclusion of three additional cases [25]. Skewness and kurtosis values for all variables fell within the acceptable range of -1 to +1. Exploratory factor analysis (EFA) was conducted using principal component analysis and varimax rotation. Models obtained from confirmatory factor analysis (CFA) were tested using the following fit indices [[25], [26], [27]]: 1) the model chi square divided by the degree of freedom (χ^2^/ df; this value should be smaller than 2.00; the lower this value is, the better the fit), 2) the comparative fit index (CFI; this value should be higher than 0.95 for a good fit; the higher this value is, the better the fit), 3) the Tuker-Lewis Index (TLI, a value above > .90 indicates an acceptable fit), 4) the standardized root mean square residual (SRMR; this value should be lower than 0.08 for a good fit; the lower this value, the better the fit), 5) the root mean square error of approximation (RMSEA; this value should be 0.08 or lower; the lower the value is, the better the fit). Statistical analyses were conducted using JASP 0.19.

## 3 Results

### 3.1 Participants’ characteristics and descriptive statistics

The final sample included 442 participants aged between 18 to 77 (*M* = 32.45, SD = 12.50) including 363 (82.13%) respondents declaring themselves as female, 74 (16.74%) as male and 5 (1.13%) as non-binary. Detailed demographic characteristics are presented in Table 1. Descriptive statistics are shown in Table 2. The female subsample was statistically significantly younger than the male subsample. Females scored higher than males on measures of general worry (PSWQ), general anxiety and stress (DASS), and anxiety about climate change (CCAS). However, there were no significant differences between males and females in terms of depression and total scores on the CCWS.

**Table 1:** Demographic characteristics of the sample (*N* = 442)

Mean age (SD)	32.45 (12.50)	
	*n*	%
Gender		
Female	363	82.13
Male	74	16.74
Non-binary	5	1.13
Marital status		
Single	224	50.68
As a couple	218	49.32
Education		
High school or less	2	0.45
Undergraduate degree	276	62.44
Graduate degree or above	164	37.10
Occupational category		
Student/vocational training	199	45.02
Employee, intermediate/high	26	5.88
Employee, low	191	43.21
Retired	8	1.81
Unemployed	18	4.07

**Table 2:** Descriptive statistics, and Student's *t*-test between male and female subsamples (*N* = 442)

	*M* (SD)				
	Total sample	Female	Male	*t*	*p*
	*N* = 442	*n* = 363	*n* = 74		
CCWS	28.94 (9.69)	29.38 (9.60)	27.08 (9.91)	1.87	.063
CCAS	52.59 (13.12)	53.38 (13.12)	49.15 (12.62)	2.54	**.011**
PSWQ	51.30 (12.73)	52.81 (12.10)	44.47 (13.50)	5.29	**< .001**
DASS depress.	12.49 (11.21)	12.91 (11.29)	11.16 (10.78)	1.22	.23
DASS anxiety	11.97 (9.76)	12.69 (10.07)	8.87 (7.51)	3.10	**.002**
DASS stress	17.72 (10.33)	18.35 (10.38)	14.97 (9.42)	2.59	**0.01**
Age	32.45 (12.50)	31.76 (12.10)	35.87 (14.08)	-2.59	**0.01**

Significant differences between female and male subsamples are bolded

CCAS Climate Change Anxiety Scale, CCWS Climate Change Worry Scale

DASS Depression Anxiety Stress Scales, PSWQ Penn State Worry Questionnaire

Reliability analysis showed strong internal consistency of the CCWS, with both Cronbach's alpha and McDonald's omega yielding values of 0.91. Regarding other scales used in this study, they displayed high internal consistency as Cronbach's alpha values were 0.92 for the PSWC, 0.89 for the CCAS, 0.95 for DASS-Depression, 0.92 for DASS-Anxiety, and 0.93 for DASS-Stress.

### 3.2 Factor analysis of the CCWS

An EFA was conducted to evaluate the factor structure of the CCWS, followed by a CFA to assess the EFA results. To ensure proper methodology, the sample was divided into two equal subsamples, as EFA and CFA should not be performed on the same data set [28]. The EFA results indicated that only one factor had an eigenvalue greater than 1 (5.67), which was confirmed by parallel analysis and scree plot. These analyses thus supported a single-factor model, which explained 51% of the variance. Table 3 shows the factor loadings of the CCWS items, with all items demonstrating good to excellent loadings between 0.61 and 0.85 [25]. Table 3 also reports descriptive statistics for each of the ten items.

**Table 3:** Descriptive statistics of CCWS items (*N* = 442) and factor loading from the EFA (*n* = 221)

Item number	Item content (abbreviated)	*M* (SD)	Factor loading
1	I worry about climate change more than other people	2.74 (1.31)	0.75
2	Worries about what the future may hold	3.66 (1.25)	0.82
3	Information about climate change in the media	2.79 (1.28)	0.62
4	Worry even when effects may time away	3.31 (1.32)	0.85
5	Worry that outbreaks of severe weather	3.94 (1.22)	0.64
6	Worry so much that paralyzed to do anything about it	1.78 (1.09)	0.61
7	I worry that not be able to cope with climate change	2.84 (1.33)	0.67
8	I notice I have been worrying about climate change	2.70 (1.42)	0.81
9	Once I begin, I find it difficult to stop	1.94 (1.20)	0.77
10	Worry how climate change may affect people I care	3.23 (1.45)	0.68

A CFA was conducted to test the one-factor model of the CCWS using all ten items. The fit indices were as follows: *χ*²/df = 161.05/35, CFI = 0.895, TLI = 0.864, SRMR = 0.056, and RMSEA = 0.128. The model showed suboptimal fit, with only the SRMR meeting the threshold for a good fit. A subsequent CFA was performed using only the six items that showed strong factor loadings (above 0.7) in the EFA. The fit indices for this reduced model were: *χ*²/df = 40.47/9, CFI = 0.96, TLI = 0.93, RMSEA = 0.126, and SRMR = 0.034. This model demonstrated an overall good fit, with CFI, TLI, and SRMR meeting the thresholds for acceptable fit.

### 3.3 Convergent and divergent validity of the CCWS

Correlation coefficients between the CCWS and other measures are presented in Table 4. The CCWS showed a strong positive correlation with the measure of anxiety about climate change (CCAS; *r*² = 0.79), indicating strong convergent validity. In contrast, the CCWS showed small positive correlations with measures of general psychopathology symptoms (DASS) and general worry (PSWQ), with *r*² values ranging from 0.24 to 0.31, suggesting good discriminant validity.

**Table 4:** Pearson's correlation between variables (*N* = 442)

	CCWS	CCAS	PSWQ	DASS depress.	DASS anxiety	DASS stress
CCWS	—					
CCAS	0.79⁎⁎⁎	—				
PSWQ	0.28⁎⁎⁎	0.31⁎⁎⁎	—			
DASS depress.	0.31⁎⁎⁎	0.37⁎⁎⁎	0.62⁎⁎⁎	—		
DASS anxiety	0.24⁎⁎⁎	0.31⁎⁎⁎	0.65⁎⁎⁎	0.78⁎⁎⁎	—	
DASS stress	0.29⁎⁎⁎	0.33⁎⁎⁎	0.72⁎⁎⁎	0.78⁎⁎⁎	0.80⁎⁎⁎	—
Age	0.00	-0.02	-0.36⁎⁎	-0.27⁎⁎⁎	-0.34⁎⁎⁎	-0.29⁎⁎⁎

CCAS: Climate Change Anxiety Scale; CCWS: Climate Change Worry Scale; DASS: Depression Anxiety Stress Scales; PSWQ: Penn State Worry Questionnaire.

* *p* < .05,

⁎⁎ *p* < .01,

⁎⁎⁎ *p* < .001

To further assess the discriminant validity of the CCWS in relation to mental health symptoms, we conducted a principal component analysis on all standardized (z-scored) items from the CCWS and the DASS. The analysis aimed to evaluate how distinctly these scales measure their intended constructs [14]. Two factors were extracted, namely a "CCWS factor" composed of CCWS items and a "general distress" factor containing DASS items. Table 5 shows the mean, standard deviation, and range of factor loadings for items from CCWS and DASS. These results indicated that, while CCWS items had good to excellent factor loading on the "CCWS factor" (between .62 and .83), CCWS items exhibited much lower loadings on the "general distress factor", ranging from .01 to .21. This suggested a robust discriminant validity of CCWS regarding psychopathology symptoms. On the other hand, DASS items showed consistently low factor loadings on the "CCWS factor" (between .05 and .25) and higher loadings on the "general distress factor" (range: .43 - .80) suggesting that DASS items are more closely associated with general distress rather than the construct measured by the CCWS. The distinct difference in factor loadings between CCWS and DASS items therefore showed a strong discriminant validity, confirming that these scales measures different psychological constructs.

**Table 5:** Discriminant validity: mean (*M*), standard deviation (SD) and range of factor loadings following principal component analysis of CCWS and DASS items (*N* = 442)

	CCWS factor		General distress factor	
	*M* (SD)	Range	*M* (SD)	Range
CCWS items	.74 (.07)	.62 - .83	.11 (.06)	.01 - .21
DASS items	.11 (.06)	.05 - .25	.68 (.08)	.43 - .80

CCWS: Climate Change Worry Scale; DASS: Depression Anxiety Stress Scales.

## 4 Discussion

The present study aimed to introduce a French version of the CCWS and assess its psychometric properties within a sample of French-speaking individuals. This research contributes to the growing body of literature examining psychological responses to climate change by adapting the CCWS for a new linguistic and cultural context. The findings not only support the reliability and validity of the French CCWS but also provide insight into its factor structure, convergent validity, and discriminant validity.

### 4.1 Psychometric properties of the French CCWS

The internal consistency of the French CCWS was found to be strong, with both Cronbach's alpha and McDonald's omega values exceeding 0.90, comparable to those reported in previous studies on the original and translated versions of the CCWS [11,14,17,18]. These results suggest that the French adaptation maintains the reliability of the original scale, making it a robust tool for measuring CCW in French-speaking populations.

The EFA indicated that a single-factor structure best fits the French CCWS, explaining 51% of the variance, consistent with findings from previous versions of the scale [11,14,17,18]. However, the CFA using all ten items showed suboptimal fit, particularly in terms of the RMSEA value, which exceeded acceptable thresholds. A revised CFA using six items with stronger factor loadings yielded better fit indices, particularly for the CFI and SRMR. These results align with past adaptations of the CCWS that encountered issues with certain items, such as the Italian version, which also required item removal to improve model fit [17]. The decision to reduce the scale to six items highlights a potential limitation of the original ten-item format, particularly when adapting the CCWS to different cultural or linguistic contexts. Future research may explore whether further refinements or alternative item phrasings are necessary to maintain the scale's validity across diverse populations.

### 4.2 Convergent and discriminant validity

The French CCWS showed strong convergent validity, as shown by its high positive correlation with the CCAS, a measure specifically designed to assess anxiety related to climate change. This is consistent with previous research, which has shown a close relationship between CCW and climate anxiety [17,18]. These findings suggest that while CCW may not encompass the full range of emotional, cognitive, and physiological symptoms associated with climate anxiety, the two constructs are closely related.

In terms of discriminant validity, the CCWS showed small positive correlations with general worry (PSWQ) and general anxiety and stress (DASS). This finding supports the idea that CCW is a distinct psychological construct that is not merely a reflection of general distress or psychopathology [11]. Furthermore, the PCA conducted to differentiate between CCWS and DASS items further underscored the discriminant validity of the CCWS, as items from the two scales loaded on separate factors. This supports the notion that CCW is conceptually distinct from broader measures of mental health symptoms.

### 4.3 Implications for understanding climate change worry

The present findings have important implications for understanding the psychological impacts of climate change. CCW, as measured by the CCWS, represents a more cognitive form of engagement with climate change, involving persistent thoughts about the potential consequences of environmental degradation. This cognitive engagement may serve as a precursor to more intense emotional responses, such as climate anxiety, which are associated with distressing emotions and physical symptoms [10].

The identification of CCW as a distinct construct has practical significance for public health and mental health interventions. As awareness of climate change continues to grow, individuals may experience heightened worry, which, if left unaddressed, could evolve into more severe forms of distress. Therefore, the CCWS can be a valuable tool for assessing individuals’ cognitive responses to climate change, allowing for early interventions that may prevent the development of climate anxiety or other mental health issues.

## 5 Limitations and future directions

Several limitations should be considered in this study. First, the use of convenience and snowball sampling methods may limit the generalizability of the findings. Future research should aim to replicate these results with more diverse and representative samples, including participants from different socio-economic backgrounds and geographic regions. Additionally, while the reduced six-item version of the CCWS showed better fit in the CFA, this modification warrants further investigation. It remains unclear whether these changes reflect cultural differences in how CCW is expressed or if they are specific to the sample used in this study. Cross-cultural comparisons between different versions of the CCWS could help clarify whether a standardized version of the scale can be used across various populations, or if cultural adaptations are necessary. Another limitation is the gender imbalance within the participant sample, with a significant skew toward female participants (82.1%). This may influence the generalizability of the results, as responses might vary with a more gender-balanced sample. Future studies could benefit from recruiting a more gender-diverse population to determine whether the psychometric properties and observed relationships remain consistent across genders or exhibit differences that could impact the overall findings.

Finally, future research could explore the longitudinal relationship between CCW and climate anxiety. Given that CCW is thought to be a cognitive precursor to climate anxiety, longitudinal studies could assess whether individuals who report higher levels of CCW are more likely to develop anxiety symptoms over time. Such research would provide valuable insights into the psychological pathways linking climate change worry, anxiety, and broader mental health outcomes.

## 6 Conclusion

In summary, this study introduces a French adaptation of the CCWS and provides evidence of its robust psychometric properties. The findings contribute to our understanding of CCW as a distinct psychological construct, with important implications for mental health research and intervention. As climate change continues to pose significant challenges to public health, tools like the CCWS are essential for assessing and addressing the psychological impacts of environmental threats across diverse cultural contexts.

## Funding sources

This research received no specific grant from any funding agency in the public, commercial, or not-for-profit sectors.

## CRediT authorship contribution statement

**Sarah Shepherd:** Writing – review & editing, Methodology, Data curation, Conceptualization. **Patrick Raynal:** Writing – review & editing, Writing – original draft, Visualization, Formal analysis. **Myriam Guedj:** Writing – review & editing, Supervision, Methodology, Conceptualization.

## Declaration of competing interest

The authors declare that they have no known competing financial interests or personal relationships that could have appeared to influence the work reported in this paper.
